# The strengths and complexities of European registries concerning paediatric kidney transplantation health care

**DOI:** 10.3389/fped.2023.1121282

**Published:** 2023-03-22

**Authors:** Loes Oomen, Liesbeth L. De Wall, Kai Krupka, Burkhard Tönshoff, Tanja Wlodkowski, Loes FM Van Der Zanden, Marjolein Bonthuis, Ilse D. Duus Weinreich, Linda Koster-Kamphuis, Wout FJ Feitz, Charlotte MHHT Bootsma-Robroeks

**Affiliations:** ^1^ Division of Paediatric Urology, Department of Urology, Radboudumc Amalia Children’s Hospital, Nijmegen, Netherlands; ^2^ CERTAIN Registry, Department of Paediatrics I, University Children's Hospital Heidelberg, Heidelberg, Germany; ^3^ ERKReg, Division of Paediatric Nephrology, Centre for Pediatrics and Adolescent Medicine, University of Heidelberg, Heidelberg, Germany; ^4^ Department for Health Evidence, Radboudumc, Nijmegen, Netherlands; ^5^ ESPN/ERA Registry, Department of Medical Informatics, Amsterdam UMC Location University of Amsterdam, Amsterdam, Netherlands; ^6^ Department Quality of Care, Amsterdam Public Health, Quality of Care, Amsterdam, Netherlands; ^7^ Department of Clinical Medicine, Scandiatransplant, Aarhus University Hospital, Aarhus, Denmark; ^8^ Department of Paediatric Nephrology, Radboudumc Amalia Children’s Hospital, Nijmegen, Netherlands; ^9^ Department of Paediatrics, Paediatric Nephrology, University of Groningen, University Medical Centre Groningen, Beatrix Children’s Hospital, Groningen, Netherlands

**Keywords:** kidney transplantation, children, registries, European union, data collection

## Abstract

**Introduction:**

Patient data are increasingly available in (multi)national registries, especially for rare diseases. This study aims to provide an overview of current European registries of paediatric kidney transplantation (PKT) care, their coverage, and their focus. Based on these data, we assess whether the current status is optimal for achieving our common goal: the optimalisation of health care.

**Methods:**

A list of all PKT centres within the European Union (EU) as well as active PKT registries was compiled using existing literature and the European Platform on Rare Disease Registration. Registry staff members were contacted to obtain information about the parameters collected and the registry design. These data were compared between registries.

**Results:**

In total, 109 PKT centres performing PKT surgery were identified in the 27 EU Member States. Currently, five European PKT registries are actively collecting data. In 39% of these centres, no data were registered within any of these five existing international registries. A large variety was observed in the number of patients, centres, and countries involved in the registries. Furthermore, variability existed regarding the inclusion criteria, definitions used, and parameters collected. Collection of perioperative urologic data are currently underrepresented in the registries.

**Discussion:**

Currently, multiple registries are collecting valuable information in the field of PKT, covering the majority of PKT centres in Europe. Due to a large variety in the parameters collected as well as different focuses, data collection is currently fragmented and suboptimal; therefore, the current existing data are incomplete. In addition, a considerable proportion of the transplantation centres do not enter data in any international registry. Combining available information and harmonising future data collection could empower the aim of these registries—namely increasing insights into the strengths and potential of current care and therefore improve healthcare

## Introduction

Over the course of the 20th century, the role of patient registries began to evolve in health care. While the first registry was reported in 1856, the use and development of registries majorly intensified with the advent of the digital era (1, 2). The use of registries or health databases is increasingly common in both clinical practice and research for collecting patient data in a systematic manner (3). Registries allow benchmarking, provide insights for evaluating practice patterns, and are a critical resource for clinical research (3, 4). The content can differ depending on the aim and the scope of the registry (3, 5, 6).

Registries are particularly helpful for rare conditions such as paediatric kidney transplantation (PKT), of which only 500 to 600 transplants are performed yearly in Europe compared to over 21,000 in adults (7–12). Registries enable the collection of high-quality data with longitudinal follow-up, which can, even in these relatively small populations, provide sufficient statistical power to identify prognostic factors in graft and patient survival, complication rates, and associations.

The European Commission developed a platform for registries on rare diseases called the European Rare Disease Registry Infrastructure (ERDRI) (10); however, not all PKT registries are findable through this platform. In addition, an overview of information on these registries is lacking, including their coverage of patients and the contents of the data collected. As each registry is expected to have a particular focus, the collected data differ between registries. In addition to the fragmentation of patient data spread over multiple registries that are hard to find, the different parameters collected make it hard to combine data from the identified registries. Therefore, reflecting on their use and goals could lead to the maturation of these registries and improve the efficiency of data collection in the future. In this era of promising technological advancements, such as automatic data extraction from medical files, it would be of particular value to evaluate current registry activities within the European Union (EU) as well as to develop a shared vision regarding the optimal use of patient data.

Therefore, the aim of this review is to provide an overview of PKT registries within the EU as well as their overlaps and differences. Based on these data, we discuss the pearls and pitfalls of registries to identify opportunities for collaboration in the future.

## Methods

A stepwise approach was used to conduct a scoping review of the existing registries and their coverage. The three steps are separately described in the following three subsections.

### Step 1: PKT centre identification

EU centres that perform PKT were identified by means of information available on all European national registries and transplantation societies from the Global Observatory on Donation and Transplantation (13). Centres were included if they were located in one of the 27 EU Member States and performed kidney transplantation surgery in patients aged below 18 years.

In addition, chairmen of each national society as well as national registry representatives known by the European Society for Paediatric Nephrology (ESPN) and European Renal Association (ERA) were contacted by email. They were requested to provide a list of centres that perform PKT in their nation (14).

Each of the identified centres were emailed to verify if they actually performed paediatric kidney transplantation surgery (included in this study) or were only performing the post-transplantation follow-up (excluded from this study). Both centres that are acknowledged as national expertise centres by their national government and centres that were not acknowledged as national expertise centres were included.

To determine the concentration of centres, we calculated the number of paediatric inhabitants per PKT for each country using the relevant demographic data from Eurostat (15).

### Step 2: PKT registry identification

A list of currently active registries on PKT centres in multiple European countries was compiled using ERDRI and information gathered from the aforementioned chairmen of national societies and national registry representatives (10).

A literature search was added as an extra control to complete the list. It was a comprehensive literature search based on the MESH terms “Kidney Transplantation”, “Children”, “Paediatrics”, “Registry”, and “European”. The search was conducted in the PubMed, EMBASE, Cochrane, and MEDLINE databases. We screened abstracts to select: dedicated PKT registries within the EU.

For each included registry, information regarding the patient enrolment criteria, data collection methods, founding year, funding, population coverage, and registry design was gathered. Registries that did not distinguish paediatric data were excluded.

### Step 3: parameter collection

All datapoints collected by the registries were subclassified into “pre-transplantation”, “transplantation”, “post-transplantation”, and “logistic (e.g., the centre code, informed consent and information on the person providing the data)” parameters and subdivided into 13 relevant topics (Supplementary Appendix S2). The numbers of parameters per topic were compared among registries, as was the handling of missing data.

## Results

### Paediatric transplantation centres

**Figure 1:**
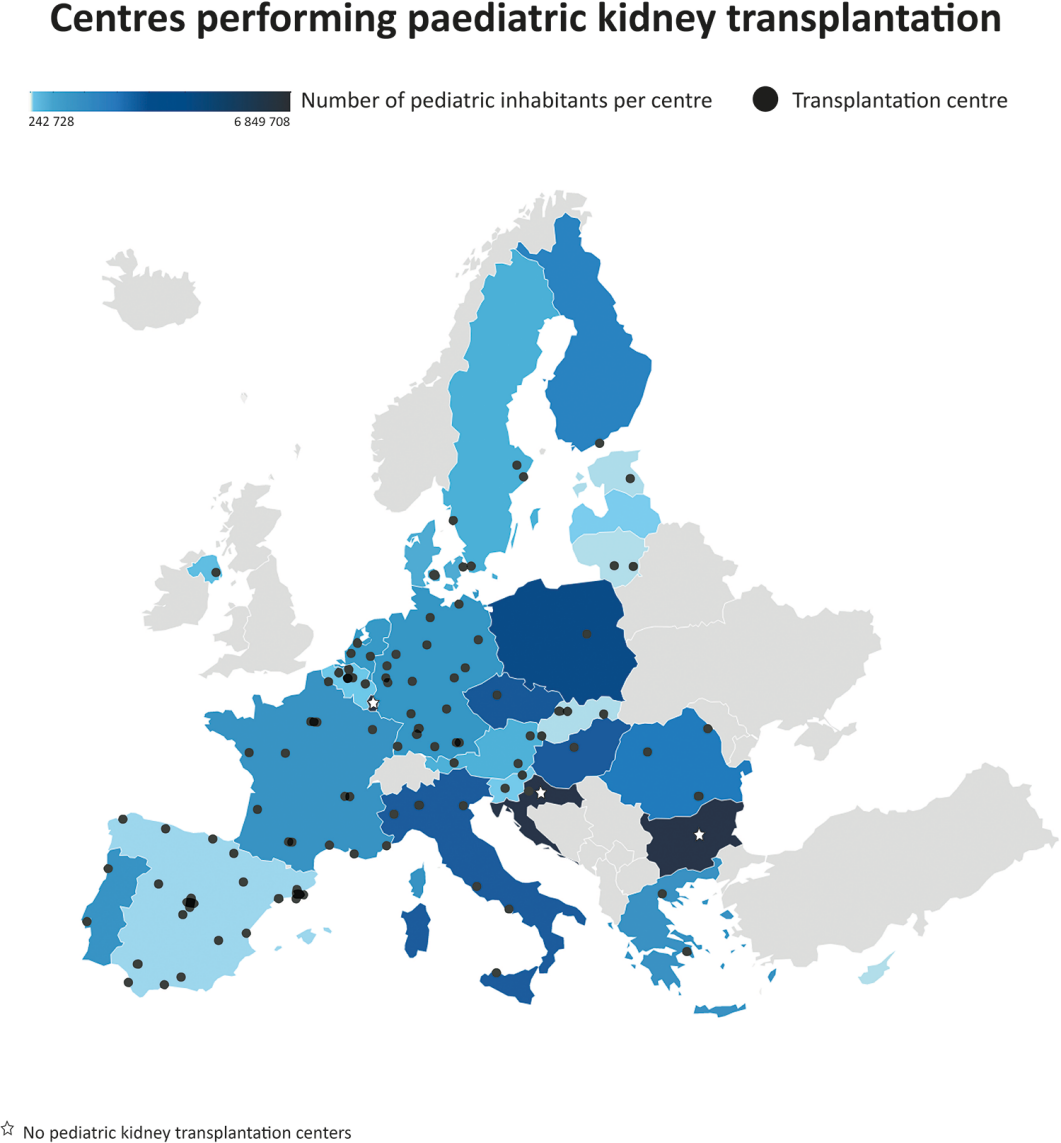
Choropleth of Europe depicting country-wise heat distribution based on the paediatric population per kidney transplantation centre. Centres (*N* = 109) that performed paediatric kidney transplantation surgery in 27 European countries. Both certified and non-certified centres are included.

National coordinators of all 27 EU member states provided information on the PKT centres in their country (Supplementary Appendix S1). In total, 117 centres were named as transplantation centres, of which 109 centres were actually performing paediatric kidney transplantation surgery. Figure 1 shows these transplantation centres, this figure indicates that the centralisation of care varies widely. Whereas in some countries all PKTs are performed in only one centre (e.g., Finland), in other countries they are scattered over more than 20 centres (e.g., Spain).

When corrected for the number of paediatric inhabitants, Cyprus was found to have the most PKT centres. Bulgaria, Croatia, Luxembourg, and Malta have no PKT centres. Patients requiring a kidney transplantation are mostly referred to centres in Austria (Croatia), Belgium (Luxembourg), Turkey (Bulgaria), and the United Kingdom (Malta).

In total, six PKT registries were identified within Europe, of which four were findable through the ERDRI. Two registries, the Collaborative Transplant Study (CTS) registry and the registry of Eurotransplant, collect data on both paediatric and adult kidney transplant recipients. Since one registry (ERN TransplantChild) was still under development, the data of five registries were included in our analysis (Table 1). A large variety was observed in the number of patients, centres, and countries involved in the different registries. For example, the number of centres actively contributing to a registry ranged from 7 to 46 and the geographical distribution differed. There were 9 centres that delivered data to the registry but did not do the surgery themselves. Besides, ESPN/ERA Registry is a population-based registry that works with regional or national coordination centres that cover data from multiple centres in that region. Moreover, variability existed with respect to the inclusion criteria for enrolment in the registries. Patients could be enrolled at the moment of diagnosis, the start of dialysis, or the moment of kidney transplantation. Furthermore, the definition of “paediatric patients” was found to differ between these registries—from under 16 years of age (Scandiatransplant) and “when treated in a paediatric department” (CERTAIN, ESPN/ERA) to <19 years old (ERN eUROGEN). In most registries, the data collection occurred at least once a year.

**Table 1:** Overview of European registries on paediatric kidney transplantation.

Registry	Funding body	Founding year	Centres involved (N)^a^	Countries involved (N)^a^	Patients registered (N)	Parameters collected (N)	Geographical distribution of centres
**CERTAIN (9)** ^b^	GPN, ESPN	2011	46	15	3,492	355	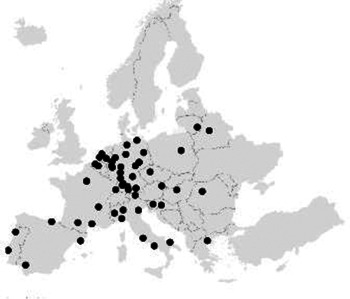
**ERKReg (8)** ^b^	ERN ERKNet	2019	18	9	1,080	52	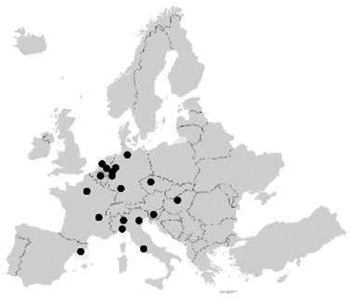
**ERN eUROGEN^b^ Registry**	ERN eUROGEN	2022	13	8	30	60	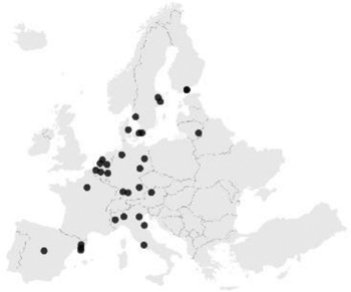
**ESPN/ERA registry (16)**	ESPN and ERA	2007	19^c^	19	10,867	120	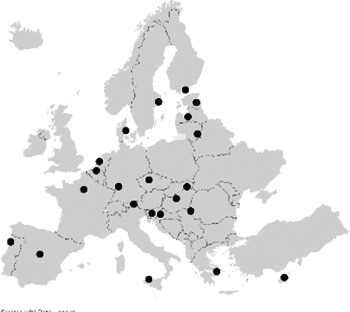
** Scandia-transplant (17)**	Scandia-transplant	1994	7	4	989	76	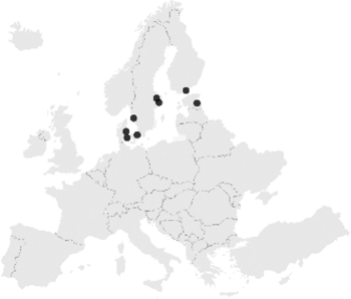

CERTAIN, Cooperative European Paediatric Renal Transplant Initiative; ERKReg, European Rare Kidney Disease Registry; ERKNet, European Rare Kidney Disease Reference Network; ERN, European Reference Network; ESPN/ERA, European Society for Paediatric Nephrology and European Renal Association; GPN, Society for Paediatric Nephrology in Austria, Germany, & Switzerland.

^a^ Centres performing PKT surgery in the 27 EU countries.

^b^ Registered in the European Directory of Registries (ERDRI).

^c^ Only the coordinating centres (one per country) are shown. The ESPN-ERA has selected one national coordinating centre to provide data of all centres in their country. These 21 coordinating centers provide data of approximately 76 centres (in total 109 PKT centers in the EU were identified) are participating *via* 21 coordinating centres.

In addition, the definitions and registration of the disease causing kidney failure were found to differ among registries. This is recorded as Orphacodes (ERKReg), or ERA disease codes (CERTAIN, ESPN/ERA, Scandiatransplant), or using an internal bespoke coding system (ERN eUROGEN).

Regarding missing data, all registries were found to have a minimal number of mandatory parameters that must be entered. Whereas ERKReg, ERN eUROGEN, and Scandiatransplant have compulsory data entry for all parameters (and therefore no missing data), CERTAIN and ESPN/ERA use a minimally required data set in combination with an extended data set that is optional.

Regarding the reward of data entry, policies also differ between registries. Some of the registries do not reimburse the centres for data entry, while others pay hospitals either per included patient or per year of activity. All registries provide data upon request (*via* an application procedure) and ask their participants if they give permission to be contacted for future research.

Although many of the identified PKT centres participated in one (30% of PKT centres) or more (31% of centres) of the identified European registries, 37% of the PKT centres only provided their data to a national registry and 2% did not participate in any registry. No centre enters data in all five registries (Supplementary Appendix S3).

### Collected parameters

**Figure 2:**
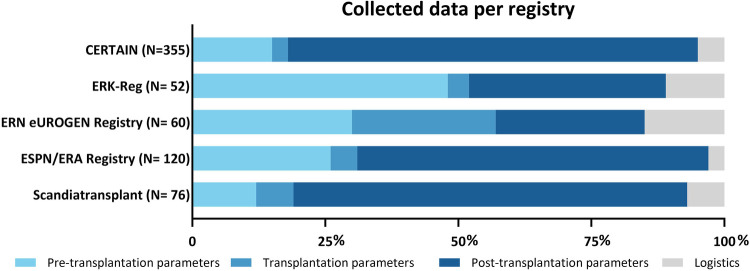
Distribution of collected datapoints by multinational European registries on paediatric kidney transplantation; *N* = number of parameters registered. CERTAIN, Cooperative European Paediatric Renal Transplant Initiative; ERKReg, European Rare Kidney Disease Registry; ERN, European Reference Network; ESPN/ERA, European Society for Paediatric Nephrology and European Renal Association.

As presented in Table 1, the comprehensiveness of the registries was found to vary greatly. Figure 2 depicts the variability in the focus of the registries (in the standard dataset). Whereas CERTAIN, ESPN/ERA Registry, and Scandiatransplant mainly focus on post-transplantation data, ERKReg has a relative focus on pre-transplantation parameters and the ERN eUROGEN registry covers all areas evenly.

**Figure 3:**
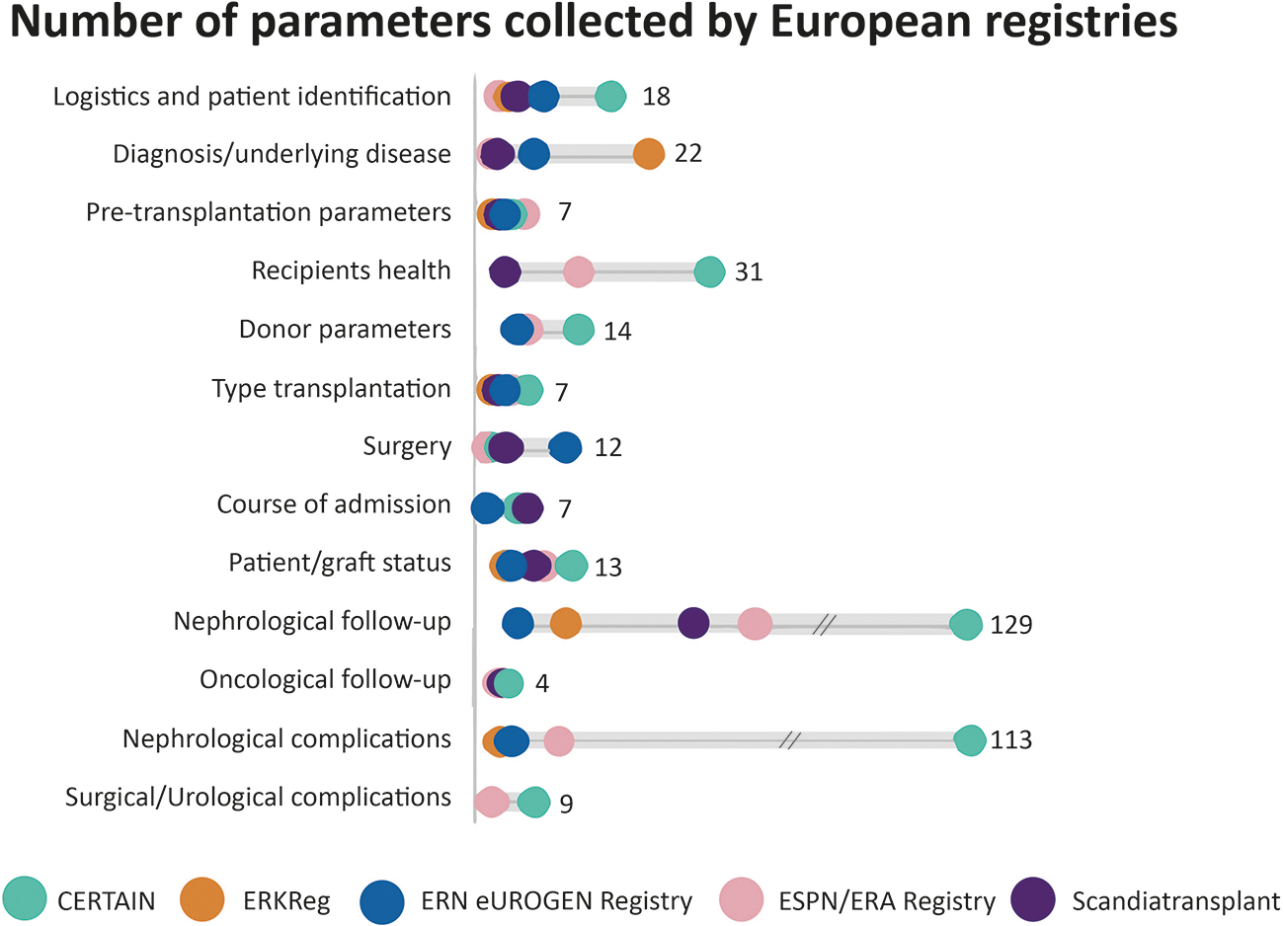
Number of parameters collected per topic per multinational European registry on paediatric kidney transplantation. CERTAIN, Cooperative European Paediatric Renal Transplant Initiative; ERKReg, European Rare Kidney Disease Registry; ERN, European Reference Network; ESPN/ERA, European Society for Paediatric Nephrology and European Renal Association.

In more detail, when subdivided into the 13 subtopics, a considerable overlap was observed, despite each registry having its own focus (Figure 3).

ERN eUROGEN Registry has, compared with the other registries, a relatively high number of “surgery” parameters, whereas ERKReg collects relatively many data on the underlying disease causing kidney failure. Perioperative urologic data (e.g., the use of stents) and information on patient-reported outcomes are currently lacking in the standard datasets of all registries.

## Discussion

In total, 109 centres in the EU perform PKT surgery, and 61% of these PKT centres participate in at least one of the five European registries. These registries vary in terms of contributing centres (7–46 centres), patients included (30–3,492), and the number and nature of collected parameters.

Worldwide, the number of registries and publications based on registries is increasing, as confirmed by an overview of kidney failure registries (3). The benefits of registries, especially in rare diseases, were discussed before, and the current PKT registries provide highly useful information that is crucial for patient management and clinical research. This enables the evaluation of current care, which is especially important since we observed large differences in the centralisation of care between countries. In addition, these data collected could provide evidence for international guidelines and clinical support tools.

However, entering data into a registry on a regular basis is labour-intensive, especially when a centre is delivering data to multiple registries. Not all registries reimburse data entry, which might be a barrier to participation, whereas voluntary data submission could result in reporting bias (18). Moreover, each registry collects a limited number of parameters that are focused on a specific area of interest, resulting in incomplete datasets. At present, urological follow-up data are scarce and data on quality of life and patient perspectives are lacking in all of these registries. Moreover, a considerable proportion of the transplantation centres are not contributing data to any international registry. Overall, despite the many advantages of these databases and the useful information they provide, much information remains lacking.

Notably, the advantages of registry studies can be amplified by engaging data from multiple registries, which may enhance statistical power and enable comparisons among districts. Moreover, sharing currently available data and coordinating forthcoming data collection will increase the quality and accuracy of current registries as well as reduce the workload for clinicians, since they would not have to provide the same data to different registries.

### Challenges for collaboration

Despite the advantages of combining data, there are still multiple impediments to smooth collaboration. First, we have demonstrated a large variability in data collection processes, inclusion criteria, and definitions of parameters. Data harmonisation (i.e., merging data with varying formats and definitions) and recoding would be necessary, although it would result in less specific data and probably the loss of information (3, 19). The balance between comprehensiveness and feasibility remains challenging. Moreover, the fact that some centres are contributing their data to multiple registries might complicate combining data since this might lead to duplication.

Second, technical impediments exist to sharing data. Secure data transfer and storage are challenging in the digital era; moreover, automated data transmission is difficult because of differences in hospital IT systems and data structure.

Third, apart from the technical challenge of secure data transfer, data availability is limited by several factors, such as data protection regulations by law, health data anonymisation, and privacy policies that vary across countries (19). Currently, no clear legal framework exists for data sharing, and furthermore, not all registries require patients to sign an informed consent form. Although the General Data Protection Regulation (GDPR) was designed to harmonise these various policies, the fragmentation of ethical standards and regulations across local institutions remains a critical barrier to international collaboration (20).

### Future perspectives

To enable smooth collaboration and improve the collection of data, we identified several opportunities for the future. Ideally, all information on a specific patient that is collected in multiple registries should be linked, resulting in near-complete databases. Directly linking data from electronic patient records will enhance the quality and quantity of the data as well as decrease the burden of manual data entry. Moreover, integration software that allows one to extract data from diverse registries and combine them into a centralised registry would be highly useful. Currently, several promising developments by the EU exist, which we discuss below.

First, the Secure Privacy-preserving Identity management in Distributed Environments for Research (SPIDER) tool was designed by the JRC. It generates pseudonyms for patients and allows the linking and transfer of data across registries without revealing patients' identities (21). A similar system was developed in Germany and is now successfully implemented (22).

Second, the ERDRI attempts to provide an overview of all European registries on rare diseases, including a description and their main characteristics (ERDRI.dor). In addition, the Central Metadata Repository (ERDRI.mdr) should facilitate harmonisation of data collection by reporting all collected parameters (10). In addition, there are more similar developments like the Medica Data Models Portal which might lead again to fragmentation (23).

Third, regarding data harmonisation, the development of the Common Data elements released by the EU's Joint Research Centre (JRC) has facilitated interoperability between registries. They are a set of 16 data elements to be registered by each rare disease registry across Europe, which are considered essential for further research (24–26).

Unfortunately, these instruments are not widely used by current registries.

Lastly, data on psychosocial effects and patients' perspective are scarce. Actively involving patients and patient recorded outcome measurements in the development of datasets could contribute to the optimisation of personalised health care.

### Strengths and limitations

This is the first study to have obtained an overview of the EU centres performing PKT and their contribution to international registries. It has accurately outlined the current landscape, since the included centres cover the complete EU population, and revealed the large differences in the centralisation of care. It therefore provides grounds for evaluating the effects of centralisation on quality of care as well as enables discussions on future policies. However, this overview might be biased since the centres that do not perform the transplantation surgery centres yet do participate in these registries were excluded from this study. It is unknown if the data of these patients are provided by both the transplantation centre and the centre providing post-transplantation care. Besides, data from national registries were used as a source to identify all paediatric kidney transplantations. This manner enables highest level of completeness of data. We state out that no distinction was made between expertise centres and other centres that do perform PKT.

In addition, this study has provided valuable information on the differences in data collection between the registries identified, and it is strengthened by the active contribution of representatives of all registries. Moreover, it linked both technological and legislative developments to future possibilities for optimising data collection.

However, this work is limited in terms of providing solutions for the aforementioned challenges. Since we have provided an overview of the current state of the art, we could only formulate suggestions for future work. In-depth analysis is required to formulate concrete action points. Therefore, more detailed information on the data entry process, validity of data, and ambitions of the registries would be useful. In this overview we included the standard datasets, however we know that several registries do collect extra data for specific research questions. Currently inactive registries were excluded from the analysis; however, an in-depth analysis of the reasons for this inactivity could contribute to an enhanced understanding of the challenges in data collection. Moreover, only international registries were studied, and many of the aforementioned centres that do not deliver data to these registries do deliver data to national registries. Combined adult/paediatric registries (such as Eurotransplant) and non-EU registries were excluded, although comparison with these registries might be of interest for future research. In addition registries are difficult to compare since ESPN/ERA Registry is a population-based registry, using one coordinating centre per region whereas other registries work with individual centres. The included registries do collect data from centres that are not in an EU member state, these were excluded from this overview.

## Conclusion

In total, 61% of the 109 PKT centres in the EU are joined with one of the five international PKT registries. Great efforts are made to collect valuable information on their paediatric kidney transplant recipients. However, due to the large variety in the parameters collected as well as the different focuses, data collection is currently fragmented and suboptimal. To optimise the use of registries, future data collection could benefit from harmonisation and the coordination of joint actions.

## Data Availability

The original contributions presented in the study are included in the article/**Supplementary Material**, further inquiries can be directed to the corresponding author/s.
