# Supplementary material for: The strengths and complexities of European registries concerning paediatric kidney transplantation health care
Source: Front Pediatr. 2023 Mar 22;11:1121282. doi: 10.3389/fped.2023.1121282 (PMC10073744; doi:10.3389/fped.2023.1121282)
Supplement: Supplementary file 1 [file Datasheet1.pdf]

Appendix 1 – Countries in Europe

Table 2 . An overview of all European Union member states, the number of centres that are performing PKT surgery and the number of inhabitants <18 years in January 2022.

	Complete response national registry staff member	Number of PKT centers	Pediatric inhabitants
**Austria**	Yes	3	1 577 640
**Belgium**	Yes	6	2 368 850
**Bulgaria**	Yes	0	1 208 260
**Croatia**	Yes	0	704 718
**Cyprus**	Yes	1	242 728
**Czech Republic**	Yes	1	2 019 811
**Denmark**	Yes	2	1 151 319
**Estonia**	Yes	1	259 442
**Finland**	Yes	1	1 042 331
**France**	Yes	17	13 761 119
**Germany**	Yes	19	14 180 298
**Greece**	Yes	2	1 678 096
**Hungary**	Yes	1	1 677 225
**Ireland**	Yes	1	441 108
**Italy**	Yes	6	9 325 877
**Latvia**	Yes	1	366 926
**Lithuania**	Yes	2	487 710
**Luxembourg**	Yes	0	120 754
**Malta**	Yes	0	76 446
**Netherlands**	Yes	3	6 849 708
**Poland**	Yes	1	1 592 785
**Portugal**	Yes	2	3 552 827
**Romania**	Yes	3	1 013 774
**Slovak Republic**	Yes	4	372 542
**Slovenia**	Yes	1	7 985 493
**Spain**	Yes	27	2 149 524
**Sweden**	Yes	4	2 149 524

Appendix 2 – Collected parameters

Table 3. Datapoints collected per registry

	Datapoints	ERK-REG	ESPN/ERA	CERTAIN	Scandia transplant	ERN eUROGEN
**Logistic and patients information**						
**A. Informed consent**						
	Patient's permission exists for being contacted for research purposes	**Yes**	No	**Yes**	No	**Yes**
	Patient's consent exists for his/her data to be reused for other research purposes	No	No	No	No	**Yes**
	Patient's biological sample available for research	No	No	No	No	**Yes**
	Biological sample type stored in a biobank	No	No	No	No	No
	Other type of biological sample stored in a biobank	No	No	No	No	No
**B. Patient Identification**						
	Patient ID	Yes	**Yes**	**Yes**	**Yes**	**Yes**
	First name	No	No	**Yes**	**Yes**	No
	Last name	No	No	**Yes**	No	No
	Patient European Code (SPIDER)	No	No	No	No	**Yes**
	Patient's pseudonym	No	No	No	**Yes**	**Yes**
	Healthcare provider	No	No	No	**Yes**	**Yes**
	Patient's date of birth	**Yes**	**Yes**	**Yes**	**Yes**	**Yes**
	Patient's sex at birth	**Yes**	**Yes**	**Yes**	No	**Yes**
	Nationality (Iso code)	No	No	**Yes**	No	No
	Ethnic Origin	**Yes**	**Yes**	**Yes**	No	No
	Residence country (ISO code)	No	No	**Yes**	No	No
	Migration background	No	No	**Yes**	No	No
	Treating Center	**Yes**	No	**Yes**	**Yes**	No
**C. Other relevant information**						
	Notes about patient's follow-up	No	No	**Yes**	No	No
	Vaccines after transplantation	No	No	No	No	No
	Has the patient joined a new prospective study since the last visit?	No	No	**Yes**	No	No
	Name study	No	No	**Yes**	No	No
	Type	No	No	**Yes**	No	No
	Study medication	No	No	**Yes**	No	No
	Blinded study	No	No	**Yes**	No	No

	Inclusion date	No	No	**Yes**	No	No
**Pre-transplantation parameters**						
**D. Diagnosis**						
**D1. Diagnosis General**	Age at which symptoms/signs first appeared	**Yes**	No	No	No	**Yes**
	Date first presentation to center	**Yes**	**Yes**	No	No	**Yes**
	Renal diagnoses established	**Yes**	No	No	**Yes**	**Yes**
	Date diagnosis established	**Yes**	No	No	No	**Yes**
	Select the diagnosis coding ontology					
	Primary renal disease code (PRD)	No	**Yes**	**Yes**	**Yes**	No
	ICD-10 Diagnosis description	No	No	No	No	No
	ORDO Description	No	No	No	No	No
	Describe others ontological classification	**Yes**	No	**Yes**	**Yes**	**Yes**
	Genetic disease	**Yes**	No	No	No	No
**D2. Establishing diagnosis**	How was diagnosis established?					
	Date immunohistochemistry	**Yes**	No	No	No	No
	Conclusion immunohistochemistry	**Yes**	No	No	No	No
	Imaging	**Yes**	No	No	No	No
	Date kidney biopsy	**Yes**	No	No	No	No
	Diagnosis kidney biopsy	**Yes**	No	**Yes**	No	No
	Immunohistochemical analysis on skin biopsy	**Yes**	No	No	No	No
	Confocal analysis on skin biopsy	**Yes**	No	No	No	No
	Date of genetic screening	**Yes**	No	No	No	**Yes**
	Was a causative gene abnormality identified?	**Yes**	No	No	No	**Yes**
	Receipt date of genetic results	**Yes**	No	No	No	**Yes**
	Methods	**Yes**	No	No	No	No
	Inheritance	**Yes**	No	No	No	No
	Affected gene	**Yes**	No	No	No	No
	Zygosity	**Yes**	No	No	No	No
	Mutation	**Yes**	No	No	No	No
	Other methodologies	**Yes**	No	No	No	No
**E. Pre-transplantation treatment**						
**E1. Waiting list**	Date of inclusion on transplant waiting list	No	No	No	No	No

	Medical Condition	No	No	No	No	No
**E2. Status at time of waiting list inclusion**	Motor Development:	No	No	No	No	No
	Cognitive Development:	No	No	No	No	No
	Academic Activity Level:	No	No	No	No	No
	Forward to transplant?	No	No	No	No	No
	Renal replacement therapy before transplantation?	**Yes**	**Yes**	**Yes**	**Yes**	**Yes**
	Mode of dialysis	**Yes**	**Yes**	**Yes**	No	No
	Months of renal replacement therapy before transplantation	**Yes**	**Yes**	No	**Yes**	No
	Nephrectomy	No	No	**Yes**	No	**Yes**
**E3. Re-transplantation**	Previous transplant?	No	**Yes**	**Yes**	No	**Yes**
	Date of the last previous transplant	No	**Yes**	No	No	No
	Number of transplants received	No	**Yes**	**Yes**	No	**Yes**
	Date of previous transplant lost	No	**Yes**	No	No	No
	Cause of previous transplant lost	No	No	No	No	No
	If Others, Cause of previous transplant lost description	No	No	No	No	No
**F. Recipients health**						
**F1. History**	History of diabetes	No	**Yes**	**Yes**	No	No
	Cardiac event	No	**Yes**	No	No	No
	CVA	No	**Yes**	No	No	No
	Vascular event	No	**Yes**	No	No	No
	Diabetes	No	**Yes**	No	No	No
	EBV	No	**Yes**	No	No	No
	History of smoking	No	No	**Yes**	No	No
	CMV	No	No	No	No	No
	Urological intervention before TX				No	No
**F2. Physical examination**	Length	Yes	**Yes**	No	**Yes**	No
	Weight	Yes	**Yes**	No	**Yes**	No
**F3. Laboratory findings**	Blood group	No	**Yes**	**Yes**	No	No
	HLA-A	No	**Yes**	**Yes**	No	No
	HLA-B	No	**Yes**	**Yes**	No	No
	HLA- DR	No	**Yes**	**Yes**	No	No
	Highest PRA	No	**Yes**	**Yes**	No	No
	Current PRA	No	No	**Yes**	No	No
	Creatinine before Tx	No	**Yes**	No	**Yes**	No
	eGFR before Tx	No	No	No	**Yes**	No
	HIV antibody	No	No	No	No	No

	HIV antigen	No	No	No	No	No
	HCV antibody	No	No	No	No	No
	HCV antigen	No	No	No	No	No
	HBc antibody	No	No	No	No	No
	HBc antigen	No	No	No	No	No
**F4. Vaccination schedule before transplantation**	Vaccines according to age and country schedule?	No	No	No	No	No
	Is the vaccination schedule contraindicated?	No	No	No	No	No
	Accelerated schedule before transplantation?	No	No	No	No	No
	Tetanus	No	No	**Yes**	No	No
	Diphtheria	No	No	**Yes**	No	No
	Pertussis	No	No	**Yes**	No	No
	Poliovirus	No	No	**Yes**	No	No
	Hep A	No	No	**Yes**	No	No
	Hep B	No	No	**Yes**	No	No
	Haemophiles influenzae b	No	No	**Yes**	No	No
	Pneumococcal	No	No	**Yes**	No	No
	Meningococcal	No	No	**Yes**	No	No
	Measles	No	No	**Yes**	No	No
	Mumps	No	No	**Yes**	No	No
	Rubella	No	No	**Yes**	No	No
	Varicella	No	No	**Yes**	No	No
	Rotavirus	No	No	**Yes**	No	No
	HPV	No	No	**Yes**	No	No
	Influenza	No	No	**Yes**	No	No
	Tick-borne encephalitis	No	No	**Yes**	No	No
	BCG	No	No	**Yes**	No	No
	Extended vaccination	No	No	**Yes**	No	No
	CMV-IgG	No	**Yes**	**Yes**	No	No
	EBV-IgG	No	No	**Yes**	No	No
	Pretreatment viral infections	No	No	**Yes**	No	No
	Desensitization procedure	No	No	**Yes**	No	No
**G. Donor parameters**						
**G1. General donor data**	Registration date	No	No	No	No	No
	ET donor nr	No	No	No	No	No
	Donor identity	No	No	No	No	No
	Donor age	No	**Yes**	**Yes**	No	No
	Gender donor	No	No	**Yes**	No	No
	Country citizenship	No	No	No	No	No
	Deceased donor type?	No	**Yes**	**Yes**	No	**Yes**

	Date death	No	No	No	No	No
	Cause of death donor	No	No	**Yes**	No	No
	Date of admission	No	No	No	No	No
	Date of admission ICU	No	No	No	No	No
	Date mechanical ventilation	No	No	No	No	No
	Date urine catheter	No	No	No	No	No
	Cardiac arrest	No	No	No	No	No
	Total duration cardiac arrest	No	No	No	No	No
	Hypotensive period	No	No	No	No	No
	Total duration of hypotensive period	No	No	No	No	No
	Date of last reanimation	No	No	No	No	No
	Duration of last reanimation	No	No	No	No	No
	Number of reanimations	No	No	No	No	No
**G2. Physical examination donor**	Weight donor	No	No	**Yes**	No	No
	Length	No	No	No	No	No
**G3. History donor**	Arterial hypertension donor	No	No	**Yes**	No	No
	Treatment hypertension	No	No	No	No	No
	Diabetes mellitus	No	No	No	No	No
	Treated	No	No	No	No	No
	Smoking	No	No	No	No	No
	Packyears	No	No	No	No	No
	IV drugs abuse	No	No	No	No	No
	Since	No	No	No	No	No
	Alcohol abuse	No	No	No	No	No
	Since	No	No	No	No	No
	Alcohol consumption	No	No	No	No	No
	Malignancy	No	No	No	No	No
	Malignancy specification	No	No	No	No	No
	Other pre-illness/previous medication	No	No	No	No	No
	PASS score	No	No	No	No	No
	Medication	No	No	No	No	No
	Trade name	No	No	No	No	No
	Dosage	No	No	No	No	No
	Route of administration	No	No	No	No	No
**G4. Radiology and pathology donor**	Date	No	No	No	No	No
	Type of diagnostics	No	No	No	No	No
	Findings	No	No	No	No	No
	Quality of graft explantation	No	No	**Yes**	No	No
**G5. Laboratory findings donor**	Last known renal function donor	No	No	**Yes**	No	No
	Date	No	No	No	No	No

	Hb	No	No	No	No	No
	Hematocrit	No	No	No	No	No
	Leucocytes	No	No	No	No	No
	Thrombocytes	No	No	No	No	No
	Red blood cell count	No	No	No	No	No
	Blood group donor	No	No	**Yes**	No	No
	EBV IgG donor	No	**Yes**	**Yes**	No	**Yes**
	CMV IgG donor	No	**Yes**	**Yes**	No	**Yes**
	CMV IgM	No	No	No	No	No
	HLA-A	No	**Yes**	**Yes**	No	**Yes**
	HLA-B	No	**Yes**	**Yes**	No	**Yes**
	HLA-DR	No	**Yes**	**Yes**	No	**Yes**
**Transplantation parameters**						
**H. Transplantation procedure**						
	Type of transplant	**Yes**	**Yes**	**Yes**	**Yes**	**Yes**
	Combined transplant	No	**Yes**	No	No	No
	Origin of donor kidney (which center)	No	No	**Yes**	No	No
	Date of transplantation	**Yes**	**Yes**	**Yes**	**Yes**	**Yes**
	Type of graft description	No	No	No	No	No
	Type of Liver graft	No	No	No	No	No
	Type of surgical technique in heart transplantation	No	No	No	No	No
	Type of graft and surgical technique in lung transplantation	No	No	No	No	No
	Type of Intestinal graft	No	No	No	No	No
	Type of pancreas graft/technique description	No	No	No	No	No
	AB0 incompatible?	No	No	**Yes**	No	No
	Relation donor-recipient	No	**Yes**	**Yes**	No	No
	Donor/receptor viral sero-mismatch?	No	**Yes**	**Yes**	No	**Yes**
	Type of viral sero-mismatch?	No	No	**Yes**	No	**Yes**
**I. Surgery**						
	Graft left/right	No	No	No	No	No
	Placement	No	No	No	**yes**	**Yes**
	Start time incision donor	No	No	No	No	No
	Start time incision recipient	No	No	No	No	No
	Start cold ischemia time	No	No	No	No	No
	End cold ischemia time	No	No	No	No	No
	Cold ischemia time	No	**Yes**	No	**Yes**	**Yes**
	Start reperfusion	No	No	No	No	No

	Kidney on machine	No	No	No	No	No
	Type of machine	No	No	No	No	No
	date and time kidney on machine	No	No	No	No	No
	Total Cold Ischemia Time (if pumped, include pump time): in min	No	No	**Yes**	**Yes**	No
	Warm ischemia time	No	No	**Yes**	No	**Yes**
	Explanation of previous grafts	No	No	**Yes**	No	No
	Post-operative drainage	No	No	No	No	**Yes**
	Ureteral splint after surgery	No	No	No	No	**Yes**
	Number of days ureteral splint	No	No	No	No	**Yes**
	TUC after surgery	No	No	No	No	**Yes**
	Number of days TUC	No	No	No	No	**Yes**
	SPC after surgery	No	No	No	No	**Yes**
	Number of days SPC	No	No	No	No	**Yes**
	Double J catheter after surgery	No	No	No	No	**Yes**
	Number of days Double J	No	No	No	No	**Yes**
**Post -transplantation parameters**						
**J. Course of admission**						
	Date of discharge	No	No	**Yes**	**Yes**	**Yes**
	On dialysis after transplantation	No	No	**Yes**	**Yes**	No
	Date start dialysis	No	No	**Yes**	No	No
	End of dialysis	No	No	**Yes**	No	No
	Good function graft at time of discharge	No	No	**Yes**	**Yes**	No
	Early Function of the Graft	No	No	**Yes**	**Yes**	No
	Any acute rejection episodes between transplant and discharge?	No	No	No	**Yes**	No
	Type of rejection	No	No	No	**Yes**	No
	Wound infection	No	No	No	No	No
	Serum creatinine day 0	No	No	No	No	No
	Serum creatinine day 1	No	No	No	No	No
	Serum creatinine day 2	No	No	No	No	No
	Serum creatinine day 3	No	No	No	No	No
	Serum creatinine day 4	No	No	No	No	No
	Serum creatinine day 5	No	No	No	No	No
	Serum creatinine day 6	No	No	No	No	No
	Serum creatinine day 7	No	No	No	No	No

	Number of days in hospitalization after transplant procedure:	No	No	No	**Yes**	No
**K. Status**						
**K1. Follow up**	Follow-up after transplant procedure	**Yes**	**Yes**	**Yes**	**Yes**	**Yes**
	Date of other follow-up	**Yes**	**Yes**	**Yes**	**Yes**	**Yes**
**K2. Patient status**	Patient reached adulthood	No	**Yes**	**Yes**	No	No
	Describe the cause of lost to follow up	**Yes**	**Yes**	**Yes**	**Yes**	No
	Patient's date of death	**Yes**	No	**Yes**	**Yes**	**Yes**
	Primary cause of death	No	**Yes**	**Yes**	**yes**	**Yes**
	Contributory cause of death	No	No	**Yes**	No	No
	Last creatinine before death	No	No	**Yes**	No	No
	Transplant related?	No	No	No	No	No
**K3. Graft status/ rejection**	Graft status	No	**Yes**	**Yes**	**Yes**	No
	Causes of graft dysfunction or failure:	No	**Yes**	**Yes**	No	No
	Renal replacement therapy after graft loss?	Yes	**Yes**	**Yes**	**Yes**	No
	Low compliance cause for graft loss?	No	No	**Yes**	No	No
	Date of graft rejection/graft loss	No	**Yes**	**Yes**	**Yes**	**Yes**
**L. Nephrological follow-up**						
**L1. Rejection**	Diagnostics	No	No	**Yes**	No	**Yes**
	Reason for diagnostics	No	No	**Yes**	No	No
	Type of rejection (Biopsy proven)	No	**Yes**	No	**Yes**	No
	Antibody-mediated changes	No	No	**Yes**	**Yes**	No
	Borderline changes	No	No	**Yes**	**Yes**	No
	T cell mediated rejection	No	No	**Yes**	**Yes**	No
	Interstitial fibrosis and tubular atrophy	No	No	**Yes**	**Yes**	No
	Describe pathology findings	No	No	**Yes**	No	No
	Second biopsy?	No	No	**Yes**	No	No
	Steroid bolus	No	No	**Yes**	No	No
	Antilymphocyte antibodies?	No	No	**Yes**	No	No
	Change in maintenance immunosuppression	No	**Yes**	**Yes**	No	No
	Dose increase of maintenance immunosuppression	No	No	**Yes**	No	No
	Intravenous immunoglobulin H	No	**Yes**	**Yes**	No	No
	Blood purification	No	**Yes**	**Yes**	No	No

	Outcome after rejection treatment	No	No	No	No	No
	Other(s) causes of dysfunction/failure description:	No	No	No	No	No
**L2.1 Physical examination**	Physical examination pathological findings	No	No	**Yes**	**Yes**	No
	Height (cm)	**Yes**	**Yes**	**Yes**	**Yes**	No
	Height Z-score <2	No	**Yes**	No	No	No
	Weight (kg)	**Yes**	**Yes**	**Yes**	**Yes**	No
	Obesity	No	**Yes**	No	No	No
	Under nutrition	No	**Yes**	**Yes**	No	No
	Bone age in years	No	No	**Yes**	No	No
	Puberty-Pubic hair	No	No	**Yes**	No	No
	Puberty-breast	No	No	**Yes**	No	No
	Puberty- testicular size	No	No	**Yes**	No	No
	Blood pressure (mmHg)	**Yes**	**Yes**	**Yes**	**Yes**	No
	Hypertension	No	**Yes**	**Yes**	No	No
	Z-score systolic blood pressure	No	**Yes**	No	No	No
	Z-score diastolic blood pressure	No	**Yes**	No	No	No
**L2.2 Laboratory results**	Lab test date	**Yes**	**Yes**	**Yes**	**Yes**	**Yes**
	Hemoglobin (g/L)	**Yes**	No	**Yes**	No	No
	Serum bicarbonate (mmol/L)	**Yes**	**Yes**	**Yes**	No	No
	Serum inorganic phosphorus (mmol/L)	**yes**	**Yes**	**Yes**	No	No
	Neutrophil count (x10e3/µL)	No	No	**Yes**	No	No
	Urea	No	**Yes**	**Yes**	No	No
	Lymphocyte count (x10e3/µL)	No	No	No	No	No
	Leucocyte count (urine and blood)	No	No	**Yes**	No	No
	Neutrophil count (urine and blood)	No	No	**Yes**	No	No
	Platelet count (x10e3/µL)	No	No	**Yes**	No	No
	Ph Urine and blood	No	No	**Yes**	No	No
	Total bilirubin (mg/dL)	No	No	No	No	No
	Total bilirubin (mcg/L)	No	No	No	No	No
	AST (UI/L)	No	No	No	No	No
	ALT (UI/L)	No	No	No	No	No
	GGT (UI/L)	No	No	No	No	No
	Albumin (g/dL)	No	**Yes**	**Yes**	No	No
	Creatinine (mg/dL)	**Yes**	**Yes**	**Yes**	**Yes**	No

	Cystatin C (mg/L)	No	No	No	No	No
	eGFR (mL/min/1.73me2)	**Yes**	**Yes**	**Yes**	**Yes**	**Yes**
	Ferritin	No	**Yes**	**Yes**	No	No
	CRP	No	**Yes**	**Yes**	No	No
	Total cholesterol	No	**Yes**	**Yes**	No	No
	HDL cholesterol	No	**Yes**	**Yes**	No	No
	LDL cholesterol	No	No	**Yes**	No	No
	Triglycerides	No	**Yes**	**Yes**	No	No
	Hypochromic red cells	No	No	**Yes**	No	No
	Serum iron	No	**Yes**	**Yes**	No	No
	Serum transferrin	No	**Yes**	**Yes**	No	No
	Sodium (U+B)	No	No	**Yes**	No	No
	Potassium (u+b)	No	No	**Yes**	No	No
	Chloride (u+b)	No	No	**Yes**	No	No
	Calcium	No	**Yes**	**Yes**	No	No
	PTH	No	**Yes**	**Yes**	No	No
	25-OH-vitamin D	No	No	**Yes**	No	No
	INR	No	No	**Yes**	No	No
	Urine Protein	**Yes**	No	**Yes**	**Yes**	No
	Urine albumin	No	No	**Yes**	**Yes**	No
	Spot Urine protein-to-creatinine ratio (mg/g)	**Yes**	No	No	**Yes**	No
	Spot Urine protein-to-creatinine ratio (mg/mmol)	**Yes**	No	No	**Yes**	No
	Spot Urine albumin-to-creatinine ratio (mg/g)	No	No	No	**Yes**	No
	Spot Urine albumin-to-creatinine ratio (mg/mmol)	No	No	No	**Yes**	No
	Left ventricular ejection fraction (%)	No	No	No	No	No
	FeV1 (%)	No	No	No	No	No
	FeV1 (L)	No	No	No	No	No
	Fraction exhaled nitric oxide - FeNO (ppb)	No	No	No	No	No
	Immunosuppressive trough level	No	No	**Yes**	No	No
	Immunosuppressive drug type	No	**Yes**	**Yes**	No	No
	Immunosuppressive trough level (ng/mL)	No	No	**Yes**	No	No
	Immunosuppressive trough level (mcg/mL)	No	No	**Yes**	No	No
	MMF AUC	No	No	**Yes**	No	No
	Immunosuppressive drug/metabolite level	No	No	**Yes**	No	No

	Within the target range trough level?	No	No	**Yes**	No	No
	Within the target range trough level?	No	No	**Yes**	No	No
	Within the target range trough level?	No	No	**Yes**	No	No
	Trough level	No	No	**Yes**	No	No
	Method	No	No	Yes	No	No
**L2.3 Functional status during the follow-up**	Motor Development:	No	No	No	No	No
	Cognitive Development:	No	No	No	No	No
	Academic Activity Level:	No	No	No	No	No
**L3. Hospitalizations**	Required hospitalization?	No	No	**Yes**	No	No
	Number of hospitalizations since last follow-up	No	No	No	No	No
	Cause of hospitalizations	No	No	**Yes**	No	No
	Days of Hospitalizations	No	No	**Yes**	No	No
	Require Intensive care unit?	No	No	No	No	No
	Days in ICU	No	No	No	No	No
**L.4 Immunosuppressive treatment**	Immunosuppressive treatment (induction and/or initial IS)	No	**Yes**	No	**Yes**	**Yes**
	Days of induction immunosuppression	No	No	No	No	No
	Did patient take immunosuppressive treatment, since last follow-up?	No	No	**Yes**	**Yes**	No
	ATG	No	**Yes**	**Yes**	No	No
	ALG	No	**Yes**	**Yes**	No	No
	OKT3	No	No	**Yes**	No	No
	Basiliximab	No	**Yes**	**Yes**	No	No
	Belatacept	No	No	**Yes**	No	No
	Daclizumab	No	**Yes**	**Yes**	No	No
	Rituximab	No	**Yes**	**Yes**	No	No
	Cyclosporin	No	**Yes**	**Yes**	**Yes**	No
	Tacrolimus	No	**Yes**	**Yes**	**Yes**	No
	Tacrolimus delayed release	No	No	**Yes**	**Yes**	No
	Sirolimus	No	**Yes**	**Yes**	**Yes**	No
	Everolimus	No	**Yes**	**Yes**	**Yes**	No
	MMF	No	**Yes**	**Yes**	**Yes**	No
	Enteric coated mycophenolate sodium	No	No	**Yes**	**Yes**	No
	Azathioprine	No	**Yes**	**Yes**	**Yes**	No
	Prednisone	No	No	**Yes**	**Yes**	No
	Prednisolone	No	No	**Yes**	No	No

	Methylprednisolone	No	**Yes**	**Yes**	No	No
	Deflazacort	No	No	**Yes**	No	No
	Eculizumab	No	**Yes**	**Yes**	No	No
**L4.1 Per medication**	Substance	No	No	**Yes**	**Yes**	No
	Trade name	No	No	**Yes**	No	No
	Status	No	No	**Yes**	No	No
	Reason	No	No	**Yes**	No	No
	Date	No	No	**Yes**	No	No
	route of administration	No	No	**Yes**	No	No
	Dosing frequency	No	No	**Yes**	No	No
	Total period dose	No	No	**Yes**	**Yes**	No
	Dosing unit	No	No	**Yes**	**Yes**	No
**L4.2 Toxicity**	Toxicity related to immunosuppressive treatment	No	No	No	No	No
	Maintenance	No	No	No	No	No
	Other immunosuppressive treatment description	No	No	No	No	No
	Describe other type of toxicity related to IS	No	No	No	No	No
	Any compliance problem?	No	No	No	No	No
	Toxicity related to Steroids	No	No	No	No	No
	Type of toxicity related to Steroids	No	No	No	No	No
	Mycophenolic acid	No	No	No	No	No
	Toxicity related to Mycophenolic acid	No	No	No	No	No
	Type of toxicity related to Mycophenolic acid	No	No	No	No	No
	Mycophenolate mofetil	No	No	No	No	No
	Toxicity related to Mycophenolate mofetil	No	No	No	No	No
	Type of toxicity related to Mycophenolate mofetil	No	No	No	No	No
	Sirolimus	No	No	No	No	No
	Toxicity related to Sirolimus	No	No	No	No	No
	Type of toxicity related to Sirolimus	No	No	No	No	No
	Everolimus	No	No	No	No	No
	Toxicity related to Everolimus	No	No	No	No	No
	Type of toxicity related to Everolimus	No	No	No	No	No
	Cyclosporine	No	No	No	No	No
	Toxicity related to Cyclosporine	No	No	No	No	No

	Type of toxicity related to Cyclosporine	No	No	No	No	No
	Azathioprine	No	No	No	No	No
	Toxicity related to Azathioprine	No	No	No	No	No
	Type of toxicity related to Azathioprine	No	No	No	No	No
	JAK inhibitors	No	No	No	No	No
	Toxicity related to JAK inhibitors	No	No	No	No	No
	Type of toxicity related to JAK inhibitors	No	No	No	No	No
	Other IS	No	No	No	No	No
	Other immunosuppressive treatment description	No	No	No	No	No
	Toxicity related to Other IS	No	No	No	No	No
	Type of toxicity related to Other IS	No	No	No	No	No
	Describe other type of toxicity	No	No	No	No	No
	Describe toxicity related to immunosuppressive agents	No	No	No	No	No
**L.5 Other medication**	Did patient take other medications?	No	No	**Yes**	No	No
	CMV hyperimmunoglobulin	No	No	**Yes**	No	No
	Trimethoprim and sulfamethoxazole	No	No	**Yes**	No	No
	Cefaclor	No	No	**Yes**	No	No
	Trimethoprim	No	No	**Yes**	No	No
	Nitrofurantoin	No	No	**Yes**	No	No
	Cefixim	No	No	**Yes**	No	No
	Ganciclovir	No	No	**Yes**	No	No
	Valganciclovir	No	No	**Yes**	No	**Yes**
	Aciclovir	No	No	**Yes**	No	**Yes**
	Valaciclovir	No	No	**Yes**	No	No
	ACE inhibitor	No	**Yes**	**Yes**	**Yes**	No
	Angiotensine 2 blocker	No	**Yes**	**Yes**	**Yes**	No
	Beta blocking agent	No	**Yes**	**Yes**	**Yes**	No
	Calcium antagonist	No	**Yes**	**Yes**	**Yes**	No
	Diuretic	No	No	**Yes**	**Yes**	No
	Recombinant human growth hormone	No	**Yes**	**Yes**	No	No
	Darbepoetin alfa	No	No	**Yes**	No	No
	Epoetin alfa	No	No	**Yes**	No	No
	Epoetin beta	No	No	**Yes**	No	No
	Epoetin theta	No	No	**Yes**	No	No

	Epoetin zeta	No	No	**Yes**	No	No
	Methoxy-PolyethylenglycolEpoetin beta	No	No	**Yes**	No	No
	Oral iron supplementation	No	**Yes**	**Yes**	No	No
	intravenous iron supplementation	No	**Yes**	**Yes**	No	No
	Calcium base phosphate lowering agent	No	**Yes**	**Yes**	No	No
	Non-calcium base phosphate lowering agent	No	**Yes**	**Yes**	No	No
	1.25OH2VitaminD3	No	**Yes**	**Yes**	No	No
	1alfa D3	No	No	**Yes**	No	No
	sodium bicarbonate	No	No	**Yes**	No	No
	sodium citrate	No	No	**Yes**	No	No
	Detail other medications received	No	No	No	No	No
	Toxicity related to other medications	No	No	No	No	No
	Toxicity related to other medications	No	No	No	No	No
	Describe other type of toxicity related to other medications	No	No	No	No	No
	Use of ESA	No	**Yes**	No	No	No
**M. Oncological follow-up**						
**M1. General post-transplant malignancy:**	Original malignancy relapse	No	No	No	No	No
	Date of relapse	No	No	No	No	No
	Post-Tx de novo malignancy?	No	No	**Yes**	**Yes**	No
	Date of post-Tx de novo malignancy diagnosis	No	No	**Yes**	**Yes**	No
	Malignancy localization	No	No	**Yes**	No	No
	Type of malignancy	No	No	**Yes**	**Yes**	No
**M2. Post-transplant Lymphoproliferative disease (PTLD)**	PTLD?	No	**Yes**	No	**Yes**	No
	Epstein-Barr Virus associated?	No	No	No	No	No
	PTLD classification (WHO 2017)	No	No	No	No	No
	Date of PTLD diagnosis	No	No	No	No	No
	PTLD localization	No	No	No	No	No
	PTLD Treatment:	No	No	No	No	No
	Other PTLD treatment description	No	No	No	No	No
	Outcome after treatment	No	No	No	No	No
**N. Nephrological Complications**						

N.1 Infections						
**N1.1 CMV infections**	Relevant infections episodes	No	**Yes**	**Yes**	No	**Yes**
	Date of infection	No	No	**Yes**	No	No
	Infection etiology	No	No	No	No	No
	CMV	No	**Yes**	**Yes**	No	**Yes**
	CMV type	No	No	**Yes**	No	No
	CMV drug resistance	No	No	**Yes**	No	No
	Kind of mutation	No	No	**Yes**	No	No
	High dose ganciclovir	No	No	**Yes**	No	No
	Alternate therapy	No	No	**Yes**	No	No
	Alternate therapy	No	No	**Yes**	No	No
	CMV antigenemia (pp65Ag)	No	No	**Yes**	No	No
	Pos PCR	No	No	**Yes**	No	No
	Pos. CMV culture	No	No	**Yes**	No	No
	CMV inclusion bodies	No	No	**Yes**	No	No
	Histopathological evidence	No	No	**Yes**	No	No
	Fever > 38 C for last 2 days	No	No	**Yes**	No	No
	New or increased malaise	No	No	**Yes**	No	No
	Leukopenia	No	No	**Yes**	No	No
	>= 5% atypical lymphocytes	No	No	**Yes**	No	No
	Thrombocytopenia	No	No	**Yes**	No	No
	Elevation of hepatic transaminases (ALT or AST) to 2 x upper limit of normal (applicable to nonliver transplant recipients)	No	No	**Yes**	No	No
	Evidence of CMV in blood by viral culture, antigenemia or a DNA/ RNA-based assay	No	No	**Yes**	No	No
	Other cause of symptoms/ signs identified	No	No	**Yes**	No	No
	Fever > 38 C for last 2 days	No	No	**Yes**	No	No
	New or increased malaise	No	No	**Yes**	No	No
	Leukopenia	No	No	**Yes**	No	No
	>= 5% atypical lymphocytes	No	No	**Yes**	No	No
	Thrombocytopenia	No	No	**Yes**	No	No
	Elevation of hepatic transaminases (ALT or AST) to 2 x upper limit of normal (applicable to nonliver transplant recipients)	No	No	**Yes**	No	No
	Evidence of CMV in blood by viral culture, antigenemia or a DNA/	No	No	**Yes**	No	No

	RNA-based assay					
	Other cause of symptoms/ signs identified	No	No	**Yes**	No	No
	Signs and symptoms of pulmonary disease in the absence of other documented cause	No	No	**Yes**	No	No
	Evidence of CMV in blood by antigenemia or quantitative PCR	No	No	**Yes**	No	No
	Bronchoalveolar lavage (BAL) fluid by antigenemia or quantitative PCR	No	No	**Yes**	No	No
	Detection of CMV in lung tissue by immunohistochemical analysis or in situ hybridization	No	No	**Yes**	No	No
	Symptoms of upper of lower gastrointestinal disease	No	No	**Yes**	No	No
	Macroscopic mucosal lesions on endoscopy	No	No	**Yes**	No	No
	Evidence of CMV in blood or biopsy tissue by antigenemia or quantitative PCR	No	No	**Yes**	No	No
	Detection of CMV in gastrointestinal tissue by viral culture, immunohistochemical analysis or in situ hybridization	No	No	**Yes**	No	No
	Elevation of bilirubin and/ or hepatic enzymes in the absence of other documented cause of hepatitis	No	No	**Yes**	No	No
	Evidence for CMV in blood by antigenemia or quantitative PCR	No	No	**Yes**	No	No
	Detection of CMV in liver tissue by viral culture, immunohistochemical analysis or in situ hybridization	No	No	**Yes**	No	No
	CNS symptoms in the absence of other documented cause	No	No	**Yes**	No	No

	Evidence for CMV in CSF samples by quantitative PCR	No	No	**Yes**	No	No
	Detection of CMV in CNS tissue by viral culture, immunohistochemical analysis or in situ hybridization	No	No	**Yes**	No	No
	Retinitis	No	No	**Yes**	No	No
	Evidence of organ dysfunction in the absence of other documented cause	No	No	**Yes**	No	No
	Evidence of CMV in blood by antigenemia or quantitative PCR	No	No	**Yes**	No	No
	Detection of CMV in affected tissue by viral culture, immunohistochemical analysis or in situ hybridization	No	No	**Yes**	No	No
	General outcome	No	No	**Yes**	No	No
	Graft function	No	No	**Yes**	No	No
**N1.2 Other infections**	EBV	No	**Yes**	**Yes**	No	**Yes**
	BKV	No	No	**Yes**	No	**Yes**
	HIV	No	No	**Yes**	No	No
	HAV	No	No	**Yes**	No	No
	HBV	No	No	**Yes**	No	No
	HCV	No	No	**Yes**	No	No
	HEV	No	No	**Yes**	No	No
	HSV	No	No	**Yes**	No	No
	VZV	No	No	**Yes**	No	No
	Other viral infection	No	No	**Yes**	No	No
	Pneumocystis jirovecii	No	No	**Yes**	No	No
	TBC	No	No	No	No	No
	Other fungal	No	No	**Yes**	No	No
	Bacterial	No	No	**Yes**	No	No
	Site of infection	No	No	**Yes**	No	No
	Opportunistic infection	No	No	**Yes**	No	No
	Catheter related?	No	No	No	No	No
	Amount of UTI in last 12 months	Yes	No	No	No	**Yes**
	Rectal carrier of resistant bacteria	No	No	No	No	No
**N2. Renal**	Renal complications	No	**Yes**	No	No	No

**complications**	Renal complication treatment	No	No	No	No	No
	Other renal complication treatment description	No	No	No	No	No
	Outcome after treatment of renal complications	No	No	No	No	No
**N3. Cardiovascular complications**	New onset High blood pressure after transplantation?	No	**Yes**	**Yes**	No	No
	Arterial Hypertension treatment	**Yes**	**Yes**	**Yes**	No	No
	systolic blood pressure	No	**Yes**	**Yes**	No	No
	diastolic pressure	No	**Yes**	**Yes**	No	No
	doppler	No	No	**Yes**	No	No
	cuff size	No	No	**Yes**	No	No
	start date	No	No	**Yes**	No	No
	24 h mean systolic blood pressure	No	No	**Yes**	No	No
	24 h mean diastolic blood pressure	No	No	**Yes**	No	No
	24h mean MAD	No	No	**Yes**	No	No
	24h mean heart rate	No	No	**Yes**	No	No
	Day period 24 h mean systolic blood pressure	No	No	**Yes**	No	No
	Day period 24 h mean diastolic blood pressure	No	No	**Yes**	No	No
	Day period 24h mean MAD	No	No	**Yes**	No	No
	Day period 24h mean heart rate	No	No	**Yes**	No	No
	Night period 24 h mean systolic blood pressure	No	No	**Yes**	No	No
	Night period 24 h mean diastolic blood pressure	No	No	**Yes**	No	No
	Night period 24h mean MAD	No	No	**Yes**	No	No
	Night period 24h mean heart rate	No	No	**Yes**	No	No
	Other arterial hypertension treatment description	**Yes**	No	**Yes**	No	No
	Outcome after treatment	No	No	No	No	No
	Transplant-Associated Thrombotic Microangiopathy (TA-TMA)	No	No	No	No	No
	TA-TMA treatment	No	No	No	No	No
	Outcome after treatment	No	No	No	No	No
	Cardiac event	No	No	No	No	No

	CVA	No	No	No	No	No
	Vascular event	No	No	No	No	No
**N4. Growth complications**	Growth (weight and/or length/height) curves below WHO Z-Score -2?	No	**Yes**	**Yes**	No	No
	Type of growth complication	No	No	No	No	No
	When the growth problems began?	No	No	No	No	No
	Reason of growth complications	No	No	No	No	No
	Explain other reason	No	No	No	No	No
**N5. Metabolic complications**	Metabolic Complications	No	No	No	No	No
	Other metabolic Complications description	No	No	No	No	No
	Diabetes mellitus	No	**Yes**	**Yes**	No	No
	Chronic treatment DM	No	No	**Yes**	No	No
	Insulin dependent DM	No	No	**Yes**	No	No
	Anorexia	No	No	**Yes**	No	No
	Hyperparathyroidism	No	**Yes**	No	No	No
	Cushingoid habitus	No	No	**Yes**	No	No
	Date metabolic complications	No	No	**Yes**	No	No
	Outcome metabolic complication	No	No	**Yes**	No	No
	Type of Bone disease	No	No	**Yes**	No	No
	Number of fractures	No	No	**Yes**	No	No
**N6. Other complications**	Upper gastrointestinal complication	No	No	**Yes**	No	No
	Lower gastrointestinal complication	No	No	**Yes**	No	No
	Glaucoma	No	No	**Yes**	No	No
	Cataract	No	No	**Yes**	No	No
	Papilledema	No	No	**Yes**	No	No
	Fundus hypertony	No	No	**Yes**	No	No
	Gingival hyperplasia	No	No	**Yes**	No	No
	Hypertrichosis	No	No	**Yes**	No	No
	Pregnancy	No	No	No	No	No
	Striae	No	No	**Yes**	No	No
	Skin warts	No	No	**Yes**	No	No
	Acne	No	No	**Yes**	No	No
	Left ventricular hypertrophy	No	No	**Yes**	No	No
**N7. Allergies /autoimmunity**	De novo allergies following transplant?	No	No	No	No	No
	Date of allergies	No	No	No	No	No
	Describe allergies	No	No	No	No	No

	De novo Autoimmunity/Immune-Mediated disorders following transplant?	No	No	No	No	No
	Date of Autoimmunity/Immune-Mediated disorders	No	No	No	No	No
	Treatment for Autoimmunity/Immune-Mediated disorders	No	No	No	No	No
	Outcome after Autoimmunity/Immune-Mediated disorders treatment	No	No	No	No	No
	Parameter (PRA,HLA 1 antibodies HLA 2 antibodies , Scd30, isoagglutinin)	No	No	**Yes**	No	No
	Date	No	No	**Yes**	No	No
	Value	No	No	**Yes**	No	No
	Unit	No	No	**Yes**	No	No
	Donor specific antibodies	No	**Yes**	**Yes**	No	No
	Assay manufacturer	No	No	**Yes**	No	No
	Other Autoimmunity/Immune-Mediated disorders description	No	No	No	No	No
**N8. Neurological and psychiatric complications**	De novo Neurological and/or psychiatric complications following transplant?	No	No	No	No	No
	Date of Neurological and/or psychiatric complications	No	No	No	No	No
	Treatment for Neurological and/or psychiatric complications	No	No	No	No	No
	Other treatment for Neurological and/or psychiatric complications description	No	No	No	No	No
	Outcome after Neurological and/or psychiatric complications treatment	No	No	No	No	No
	Other Neurological and/or psychiatric complications description	No	No	No	No	No
**O. Surgical complications and procedures**						
	Has the patient had any surgical complications or procedures related to transplantation, since last	No	No	**Yes**	No	No

	follow up?					
	Which one?	No	No	No	No	No
	Surgical complication type	No	No	No	No	No
	Wound healing problems	No	No	No	No	No
	Vascular	No	No	No	No	No
	Describe the localization of vascular complication	No	No	No	No	No
	Ureteral obstruction	No	No	Yes	No	No
	Urine leakage	No	No	No	No	No
	Renal artery stenosis	No	**Yes**	Yes	No	No
	Avascular necrosis	No	No	No	No	No
	Thrombosis/embolism	No	**Yes**	No	No	No
	Specific surgical complications of transplantation	No	No	No	No	No
	Type of venous catheter complication	No	No	No	No	No
	Type of treatment	No	No	No	No	No
	Type of procedure (other than surgical complications treatment)	No	No	**Yes**	No	No
	Date of surgical complications or procedures	No	No	**Yes**	No	No
	Outcome after surgical complications or procedures	No	No	**Yes**	No	No
	If any other has been selected, please describe	No	No	No	No	No
	Procedures after last follow up?	No	No	**Yes**	No	No
	Describe procedures	No	No	**Yes**	No	No
	Number of procedures	No	No	No	No	No
	General outcome	No	No	**Yes**	No	No

CERTAIN: Cooperative European Paediatric Renal TransplAnt Initiative, ERK-Reg: European Rare Kidney Disease Registry, ERN: European Reference Network, ESPN/ERA: European Society of Paediatric Nephrology and European Renal Association

Appendix 3- Participation in European registries

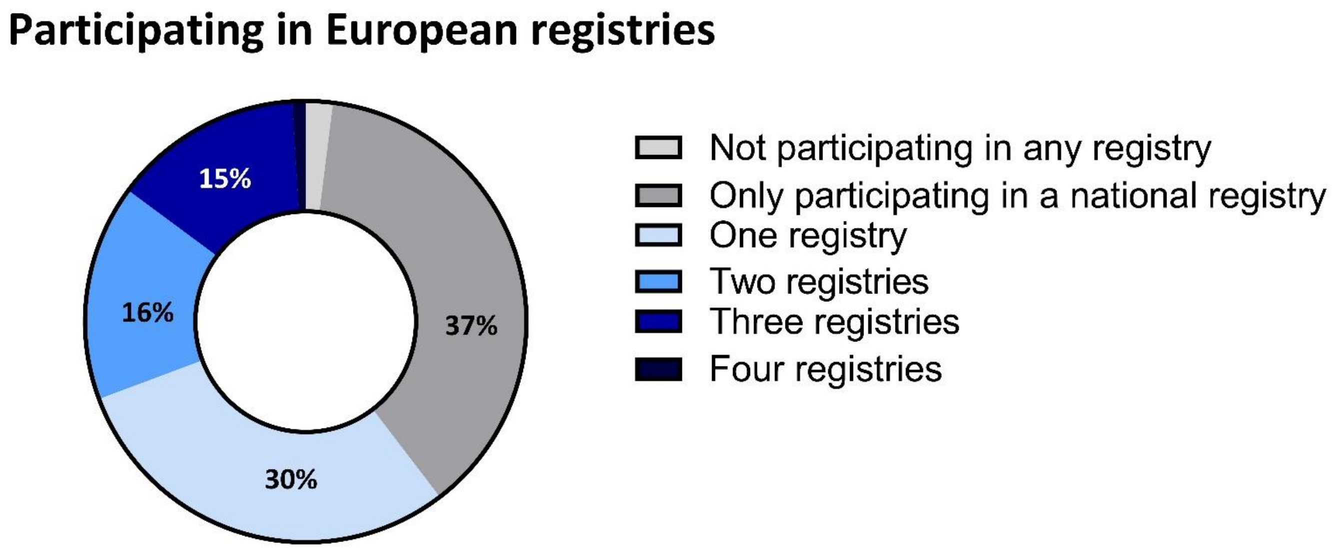

Figure 4. Percentage of centers (n=109) that are delivering data to 0,1,2,3 of 4 multinational European registries. No center delivers data to all 5 registries.
